# Recurrent priapism in the setting of cannabis use

**DOI:** 10.1186/s42238-020-0015-8

**Published:** 2020-02-13

**Authors:** Sebastian Montgomery, Kristal Sirju, Joseph Bear, Latha Ganti, John Shivdat

**Affiliations:** 1 Coliseum Medical Centers/ Mercer University, 350 Hospital Drive, 31217 Macon, Georgia United States; 2 Southeastern Urology Associates, Macon, Georgia, United States; 3 Envision Physician Services, Nashville, Tennessee, United States

## Abstract

Priapism (persistent and painful erection of the penis) is a notable urological emergency, with over 90% of those remaining erect for 24 h losing sexual function. Drug-induced priapism is common in the adult population, with intracavernosal injectables for erectile dysfunction topping the list. A variety of illicit drugs associated with priapism have been described; however, we are not aware of any other case reports showing cannabis alone as the inciting factor. Here, we present a case of a healthy 32-year-old African American man with a history of stuttering (recurrent) priapism secondary to mild cannabis substance use without comorbid substance use, licit or illicit.

## Introduction

Priapism is defined by an erection that persists for longer than four hours that is not related to sexual stimulation. It is divided into two main groups, ischemic or low-flow and non-ischemic or high-flow priapism. The majority of cases encountered in the emergency department are ischemic priapism, which results from failed relaxation of cavernosal smooth muscle (Broderick et al., 1994). To the patient, the risk of priapism is obvious, as ischemic priapism can cause serious complications, as the blood trapped in the penis is deprived of oxygen. When an erection lasts longer than four hours, this hypoxemic environment can lead to damage to the penile tissue, with notable destruction obvious at twelve hours (Spycher et al., 1986). As a result, untreated priapism can cause permanent loss of sexual function and must be treated as a urological emergency. Priapism itself has a bimodal distribution, with the majority of childhood cases involving sickle cell anemia and adult cases with known etiology involving intracavernosal injections. Drug-induced priapism has long been proposed to include PDE-5 inhibitors, anticoagulants, antihypertensives, antidepressants, alpha-blockers, and recreational drugs (most notably cocaine). We conducted a PubMed search with priapism and cannabis (cannabinoid, cannabis), limiting it to English language articles in adults. No publication year limit was imposed. No case reports were found that described priapism in the setting of cannabis use without concurrent medical disease or drug use. Of the four previously published case reports linking cannabis use to priapism, this is the first that we are aware of that excluded all other well-established causes of priapism.

## Case report

We present the case of a healthy 32-year-old African American man who presents to the emergency department with persistent erection for six hrs not related to sexual activity. Notably, the patient had been seen two weeks prior in our emergency department for a persistent erection lasting twelve hrs. At that time he underwent a needle aspiration with phenylephrine injection leading to successful detumescence. He admitted to smoking cannabis several nights per week for the past six months, including within the two hour period prior to each presenting episode of priapism. During this time, the patient had four or more episodes of a persistent erection lasting close to four hours that were self-resolving. He reported a previous full outpatient evaluation for sickle cell trait and anemia as a teenager that was negative, and denied any known relatives with sickle cell disease or trait. He admitted a history of cannabis use at age sixteen and seventeen, during which time he had recurrent priapism lasting less than four hours and never requiring medical treatment. He quit cannabis use in his twenties, and during this period did not have any episodes of priapism. He denied any history of psychiatric disease and took no prescribed or over-the-counter medications, specifically denying psychiatric medications or blood pressure medications. On physical exam, the patient was mildly hypertensive with an erect, swollen, and tender penis. A repeat needle aspiration with (1000 mcg total) of phenylephrine was completed, again resulting in successful detumescence. A urine drug screen was consistent with cannabis use without other drug use. A complete blood count revealed no anemia and a normal mean corpuscular volume. The patient was again referred to urology and internal medicine on an outpatient basis for further workup; however he was lost to follow-up in this period.

## Discussion

This case report examines the first known case of cannabis-associated priapism in a patient where all other known causes of priapism have been excluded. While cannabis use has already been noted in educational sources and textbooks as a potential cause of priapism, an electronic literature review was only able to identify four distinct cases of cannabis use coinciding with priapism, none of which were convincingly able to prove cannabis was the sole cause (Reichman, 2013). In the first two papers, the patients had cannabis use and concurrent sickle cell trait (Matta et al., 2014; Birnbaum and Pinzone, 2008). Sickle cell disease itself is the number one cause of secondary priapism in children ages five to ten years old (Banos et al., 1989). The third report notes cannabis and 3,4-methylenedioxymethamphetamine (MDMA or ecstasy) use prior to the episode of priapism, with MDMA already having been proven a cause of priapism (Tran et al., 2008). The fourth case report notes concurrent insulin dependent diabetes mellitus, cocaine use, and anabolic steroid use, with cocaine and diabetes each previously described causes of priapism (Evans et al., 2016). An additional report gives supporting evidence that synthetic cannabinoids, which are 100 times more potent activators of the same cannabinoid type 1 receptor (CB1R) as THC, can cause priapism (Ortac et al., 2018; Wiley et al., 2014). If synthetic cannabinoids can cause priapism, plant cannabis, affecting the same CB1R, would also be capable to potentiate this reaction.

In total, there are over 400 psychoactive compounds in cannabis. Of these, delta-9-tetrahydrocannabinol, or THC, is both found in the highest quantities and the primary psychoactive compound (Atakan, 2012). THC interacts with the two primary cannabinoid receptors, Cannabinoid type 1 receptor and cannabinoid type 2 receptor (CB2R). THC primarily interacts with CB1R, with its major psychoactive effects due to CB1R’s presence in the central nervous system’s basal ganglia, limbic system, hippocampus, and cerebellum; however, CB1R can also be found throughout the peripheral body, notably in the peripheral nervous system, uterus, testicular tissues, and vasculature (Russo and Guy, 2006; Pagotto et al., 2006; Pertwee, 2006). It is possible that the sympathetic blockage thought to occur as a result of cannabinoid activity limits the ability of the thoracolumbar sympathetic pathway to cause detumescence or that the now unopposed sacral parasympathetic activity that initiated the erection increases the risk for priapism (Dean and Lue, 2005). Alternatively, cannabinoids direct vascular effects could potentiate the unrelenting erection notable in priapism.

A third possible effect of more chronic cannabis use involves the thrombogenic effects caused by increased platelet activation (Randall, 2007). There is noted expression of CB1R and CB2R on platelets and during THC use there is a measurable increase in platelet expression of glycoprotein IIb-IIIa and P-selectin, resulting in greater platelet activation (Deusch et al., 2004). This culminates in a 4.8-fold increase in myocardial infarction in the 60 min after THC use (Mittleman et al., 2001). These factors together could lead to thrombotic causes of priapism, similar to that noted in sickle cell patients. Our patient has a direct, albeit circumstantial, connection between his recurrent (stuttering) priapism and cannabis use. He notes recurrent priapism when heavily using cannabis at age sixteen and seventeen. These episodes each lasted under four hours and resolved without medical intervention or medical examination. When the patient stopped using cannabis at age eighteen, his priapism resided, with no notable episodes in his twenties. He once again resumed his use of cannabis over six months ago and noted at least a dozen episodes that self-resolved in under four hours at home. The abstinence and subsequent use of cannabis were the only appreciable factors in this patient’s battle with recurrent unwanted erections.

## Conclusion

In conclusion, cannabis use is a likely cause for priapism in our patient. He had no medical history other than mild hypertension, he took no medications, and used only cannabis, supported by his urinary drug screen. Further, his history exhibited a convincing correlation between his cannabis use and his episodes of recurrent priapism. Because cannabis is the most widely used illicit substance, its link to priapism suggests it may soon become more prominent within the emergency department (Ortac et al., 2018).

## Data Availability

N/A (retrospective chart review)

## Abbreviations

CB1R: Cannabinoid type 1 receptor
CB2R: Cannabinoid type 2 receptor
ED: Emergency department
PDE: Phosphodiesterase
THC: Tetrahydrocannabinol
